# Safety of Catheter Embolization of Pulmonary Arteriovenous Malformations—Evaluation of Possible Cerebrovascular Embolism after Catheter Embolization of Pulmonary Arteriovenous Malformations in Patients with Hereditary Hemorrhagic Telangiectasia/Osler Disease by Pre- and Post-Interventional DWI

**DOI:** 10.3390/jcm10040887

**Published:** 2021-02-22

**Authors:** Guenther Schneider, Alexander Massmann, Peter Fries, Felix Frenzel, Arno Buecker, Paul Raczeck

**Affiliations:** Clinic of Diagnostic and Interventional Radiology, Saarland University Medical Center, Kirrberger Str. 1, 66421 Homburg, Germany; Alexander.Massmann@uks.eu (A.M.); peter.fries@uks.eu (P.F.); felix.frenzel@uks.eu (F.F.); arno.buecker@uks.eu (A.B.); paul.raczeck@uks.eu (P.R.)

**Keywords:** hereditary hemorrhagic telangiectasia/HHT/osler’s disease, cerebral ischemic lesions, catheter based embolization therapy, pulmonary arteriovenous malformations

## Abstract

Background. This paper aimed to prospectively evaluate the safety of embolization therapy of pulmonary arteriovenous malformations (PAVMs) for the detection of cerebral infarctions by pre- and post-interventional MRI. Method One hundred and five patients (male/female = 44/61; mean age 48.6+/−15.8; range 5–86) with pre-diagnosed PAVMs on contrast-enhanced MRA underwent embolization therapy. The number of PAVMs treated in each patient ranged from 1–8 PAVMs. Depending on the size and localization of the feeding arteries, either Nester-Coils or Amplatzer vascular plugs were used for embolization therapy. cMRI was performed immediately before, and at the 4 h and 3-month post-embolization therapy. Detection of peri-interventional cerebral emboli was performed via T2w and DWI sequences using three different b-values, with calculation of ADC maps. Results Embolization did not show any post-/peri-interventional, newly developed ischemic lesions in the brain. Only one patient who underwent re-embolization and was previously treated with tungsten coils that corroded over time showed newly developed, small, diffuse emboli in the post-interventional DWI sequence. This patient already had several episodes of brain emboli before re-treatment due to the corroded coils, and during treatment, when passing the corroded coils, experienced additional small, clinically inconspicuous brain emboli. However, this complication was anticipated but accepted, since the vessel had to be occluded distally. Conclusion Catheter-based embolization of PAVMs is a safe method for treatment and does not result in clinically inconspicuous cerebral ischemia, which was not demonstrated previously.

## 1. Introduction

Arteriovenous malformations (AVMs) in patients with Hereditary Hemorrhagic Telangiectasia (HHT; Osler’s disease) are malformations in which arteries and veins are directly connected, due to the absence of intervening capillaries [1]. The most common clinical symptoms are spontaneous and recurrent epistaxis, as well as Telangiectasias (small AVMs) on the lips, tongue, buccal mucosa, face, chest, and fingers [2]. Larger AVMs become symptomatic in the lungs, liver, gastrointestinal tract, or brain; thus, complications from severe bleeding or shunting with possible consecutive cerebrovascular incidents may occur. Pulmonary arteriovenous malformations (PAVMs) are defined as pathologic communications between pulmonary arteries and pulmonary veins, resulting in a right-to-left shunt [3,4]. Larger shunts may result in hypoxemia manifesting with dyspnea, potentially increasing the risk of paradoxical cerebral embolization [5], and, in consequence, the risk of increased morbidity and mortality. Of the approaches to treating patients with PAVMs, catheter embolization, either with coils or vascular plugs [6], is considered the treatment of choice because of its high success rate and reduced invasiveness compared to lung surgery [7,8], and because embolization more favorably respects the unaffected lung parenchyma compared to surgical resection [9].

Reperfusion or recanalization of initially successfully treated PAVMs is the most common cause of recurrence after coil embolization [10,11,12]. However, interventionalists can minimize the risk of reperfusion by using dense “packing” techniques that result in the complete cross-sectional occlusion of feeding arteries [13]. Thus, PAVM embolization with Amplatzer vascular plugs (AVP) has been shown to achieve relatively high mid-term success rates in terms of recurrence or recanalization, even in bilateral treatment [14,15,16,17]. In general, a low reperfusion rate is noted in the long-term due to late re-opening [6,18].

The incidence of stroke in patients with HHT ranges between 9 and 18% [19,20]. Although the occurrence of clinically conspicuous stroke seems to be lower in patients with PAVMs treated with embolization therapy than in patients with untreated, persistent PAVMs [19,20,21,22], little is yet known about the rate of clinically inconspicuous ischemic brain lesions associated with PAVMs. In contrast, procedure-associated, clinically inconspicuous ischemic brain lesions are common in up to 40% of patients undergoing supra-aortal endovascular procedures or neurovascular interventions, such as carotid stenting or endarterectomy [23,24].

To our knowledge, no data are yet available on the incidence of peri-interventional cerebral ischemia occurring during catheter-based embolization of PAVMs. Therefore, the aim of our investigational study was to prospectively evaluate the incidence of peri-interventional cerebrovascular incidents in patients with HHT referred for catheter-based embolization of PAVMs.

## 2. Materials and Methods

### 2.1. Patients

This single-center, prospective study observational was approved by the institutional review board. Written informed consent for both catheter-based embolization and the use of imaging data was obtained from all patients or legal guardians.

All patients included in the study suffered from HHT, confirmed either by genetic testing or, in most cases, based on Curaçao criteria [1,25]. Independently of clinical presentation and symptoms, each included patient had at least one PAVM with a feeding artery diameter of at least 2 mm diagnosed by contrast-enhanced MR angiography (CE-MRA), and in a few cases, CT imaging.

Patients were ineligible for inclusion if they had a severe allergy to iodine contrast agents, significantly impaired renal function (GFR < 15 mL/min), and/or severely impaired blood coagulation (INR > 2) or platelet count (<50.000/dL). Likewise, patients were ineligible for inclusion if they were contraindicated for MRI (e.g., for implanted cardiac pacemakers).

### 2.2. Embolization Technique

Access through the right common femoral vein was obtained after local anesthesia of the groin region. A 7F sheath was inserted and the common pulmonary artery was probed with the help of a 5F pigtail catheter and a bentson guidewire. Diagnostic pulmonary angiograms were performed to locate and visualize the PAVMs. Afterwards, using a Rosen guidewire, a Cook White Lumax guiding catheter (Cook Medical) or a coaxial system consisting of a Neuron 6F Long Sheath and a Neuron 6F Select Catheter (Penumbra) was inserted, and selective catheterization of the segmental and subsegmental pulmonary artery feeding the PAVM was performed, using the coaxial system. Guidewires which might perforate the aneurysm sac were avoided. The number and diameters of the feeding arteries of the evaluated PAVMs were identified after contrast medium injection. The PAVMs were classified as simple or complex based on the number of feeding arteries, as described elsewhere [26,27].

Depending on the size of the main feeding artery and the anatomical situation, either Nester-Coils (Cook, USA) or Amplatzer vascular plugs II/IV (St. Jude Medical) were used for embolization. These were introduced through a guiding catheter of appropriate size under a water seal. The diameter of the device was chosen to be approximately 30% larger than the size of the main feeding artery. The plug or coil was then placed as distally as possible in the feeding artery with sparing of the PAVM itself.

The choice of embolization device was made according to the length of the available landing zone, which is the distance between the PAVM and the first proximal pulmonary subsegmental artery. In the case of amplatzer vascular plugs, the position of the device was checked by Digital Subtraction Angiography (DSA) immediately after device placement. If the position of the device was deemed adequate and satisfactory, the device was released; otherwise, the device was retrieved and repositioned as necessary.

Post-embolization angiography was performed after satisfactory device placement to confirm the total occlusion of the PAVM.

Immediately before the procedure, each patient received IV injection of 2500 IE Heparin.

The number of PAVMs treated in each patient ranged from one to eight, either treated in one intervention or across multiple interventions, depending on the duration and complexity of the procedure as well as the patient’s general condition of compliance during angiography.

### 2.3. Pre- and Post-Interventional Pulmonary MRI

Pulmonary CE-MRA to evaluate PAVMs before and after intervention was performed on a 1.5 Tesla (T) magnet (Magnetom Aera, Siemens Medical Systems, Erlangen, Germany) with a 16-channel phased-array coil. The imaging protocol consisted of dynamic, time-resolved, contrast-enhanced MRA, and high-resolution, pulmonary arterial- and early venous-phase, contrast-enhanced MRA sequences.

Time-resolved MRA was performed after injection of a small contrast bolus (0.025 mmol/kg of gadobenate dimeglumine [MultiHance^™^, Bracco] or 0.05 mmol/kg of gadoteridol [ProHance^™^, Bracco]). The sequence parameters were as follows: repetition time/echo time (TR/TE) = 2.7/1.0 ms, average field of view = 40 × 29 cm, slice thickness = 1.5 mm, 140–160 slices, BW = ±113 kHz. The temporal resolution of the sequence was 3 sec/dataset with a total of 72 slices. *k*-space sampling was performed via key-hole imaging (TWIST). The true spatial resolution was 1.2 × 1.2 × 1.5 mm^3^, which was interpolated to 0.7 × 0.7 × 1.0 mm^3^ by zero-filling.

High-resolution, contrast-enhanced Angio 3D MRA was then performed using the timings established in the time-resolved study. Initially, breath-hold, non-contrast enhanced, T1-weighted, spoiled gradient recalled echo (FLASH 3D) images were acquired. The sequence parameters were as follows: TR/TE = 2.81/1.07 ms, average field of view = 40 × 29 cm, slice thickness = 1.3 mm, 140–160 slices, BW 540 kHz. The temporal resolution of the sequence was 2.2 s/dataset with a total number of up to 160 slices. The true spatial resolution was 1.3 × 1.3 × 1.5 mm^3^, which was interpolated to 1.1 × 1.1 × 1.3 mm^3^ by zero-filling. Thereafter, the identical FLASH 3D sequence was repeated after injection of 0.075 mmol/kg of gadobenate dimeglumine or 0.15 mmol/kg of gadoteridol at a flow rate of 2 mL/s at end-inspiration, followed by a flush of 30 mL normal saline [28]. The scan time varied depending on patient size and the number of slices required. Likewise, the acquisition time varied with the size of the patient and the number of phase-encoded steps needed to maintain resolution. Iterative reconstruction was applied to provide an effective acceleration factor of approximately 4.0, which also varied slightly depending on the number of slices.

The first acquisition was the arterial phase of the pulmonary circulation, and possible shunts between the bronchial arteries and pulmonary veins were also visualized during this phase. Subsequently, a second full acquisition was performed in which normal pulmonary veins were visible. For all acquisitions, patients were instructed to hold their breath at end-inspiration. The total acquisition time for the entire MRA protocol ranged between 5 and 6 min. All examinations were performed as part of the daily clinical routine. Follow-up of all interventional procedures by means of CE-MRA was performed routinely, first at 3 months post-intervention and then at yearly intervals.

### 2.4. Cerebral MRI

Cerebral MRI was performed immediately before the embolization, as well as at the 4 h and 3-month post-embolization therapy. For detection of peri-interventional cerebral ischemic lesions, T2w imaging (T2-Turbo Spin Echo [TSE], slice thickness 3 mm, TR = 5000 ms, TE = 92 ms, BW 191 kHz) and Diffusion Weighted Imaging (DWI; echo planar imaging sequence, slice thickness 5 mm, TR = 6300 ms, TE= 89 ms, BW: 1132 kHz) using three different b-values (b = 0, 400, 800) with calculation of ADC maps were performed. Any new lesion occurring between the pre- and post-interventional cerebral MRI scans on either sequence was considered a new cerebrovascular incident associated with the intervention. Additional pre- and post-contrast T1-weighted TSE, FLAIR, and susceptibility weighted sequences were acquired as part of the initial screening to rule out cerebral AVMs, including micro-AVMs.

### 2.5. Statistical Analysis

The characteristics of all participants were transcribed into software (Excel, version2011; Microsoft, Redmond, Wash) for subsequent analysis. Central tendency was measured by the mean, while range and standard deviation were used to measure the dispersion of data.

## 3. Results

Between 2008 and 2019, a total of 105 patients (male/female = 44/61; mean age 48.6 +/− 15.8 (range 5–86)) met the inclusion criteria and were included in the study. Overall, 289 PAVMs were embolized across these 105 patients. This total included 47 (16.3%) re-perfused PAVMs in 35 (33.3%) patients. A total of 871 embolization coils and 119 vascular plugs were used. No technical difficulties occurred during placement or deployment of the embolization device.

No cerebrovascular incidents directly ascribable to the embolization procedure occurred. Small, diffuse, but clinically inconspicuous acute cerebral lesions were detected in one patient (1/105; 0.95%) on DWI-MRI at 4 h after the interventional procedure, but this patient had previously undergone embolization of a vessel with tungsten coils that had corroded over time. Since re-embolization into the previously placed tungsten coils was considered necessary and unavoidable, the possibility of new cerebral emboli resulting from small particles of corroded tungsten coil released during the re-embolization was anticipated prior to the treatment. The re-embolization of this patient was successful, and no further brain lesion and no clinical symptoms of stroke were encountered over a follow-up period of 8 years.

No other patient, whether undergoing primary embolization or re-embolization, showed any signs or symptoms of cerebrovascular incidents, and no newly developed clinically inconspicuous ischemic brain lesions were observed on MRI.

**Figure 1:**
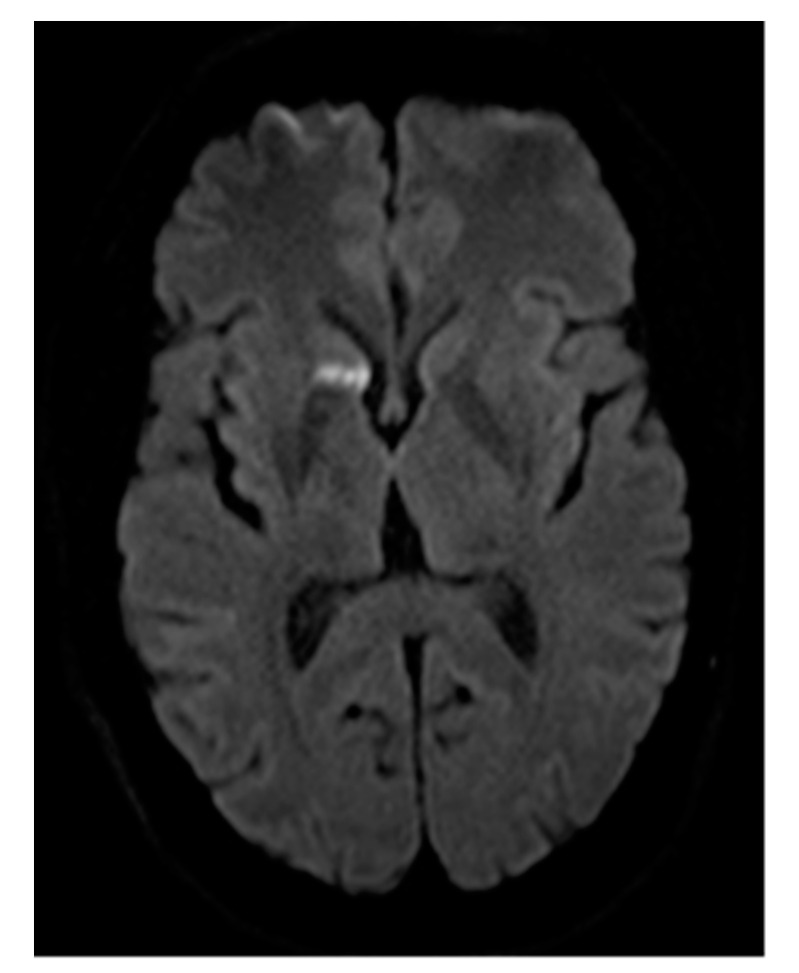
Cerebral DWI (b = 800) demonstrating subacute cerebral ischemia, in this case prior to embolization of multiple PAVMs. The newly developed lesion arose between screening for PAVM and the day of interventional therapy, but the patient did not exhibit any clinical signs or symptoms. This example highlights the importance of DWI for the detection of pre-existing cerebral ischemia, as well as peri-interventional cerebral insult.

**Figure 2:**
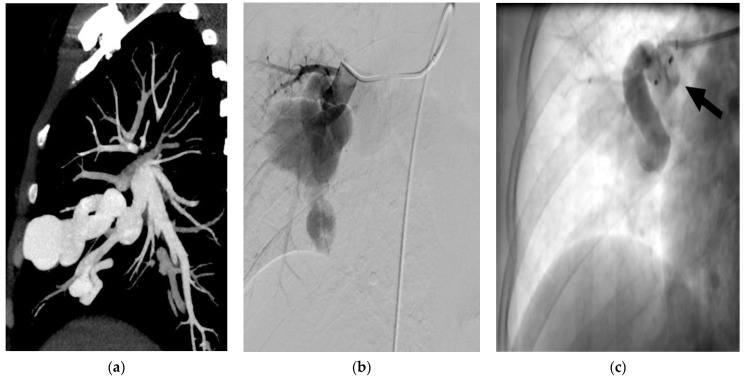
(**a**–**c**) CT of a patient, demonstrating a giant complex PAVM of the right lung (**a**). The PAVM is depicted after selective catheterization of the feeding artery on DSA after manual contrast medium injection (**b**). DSA of the PAVM directly after positioning of an amplatzer vascular plug II (arrow) with already reduced flow in the PAVM (**c**).

**Figure 3:**
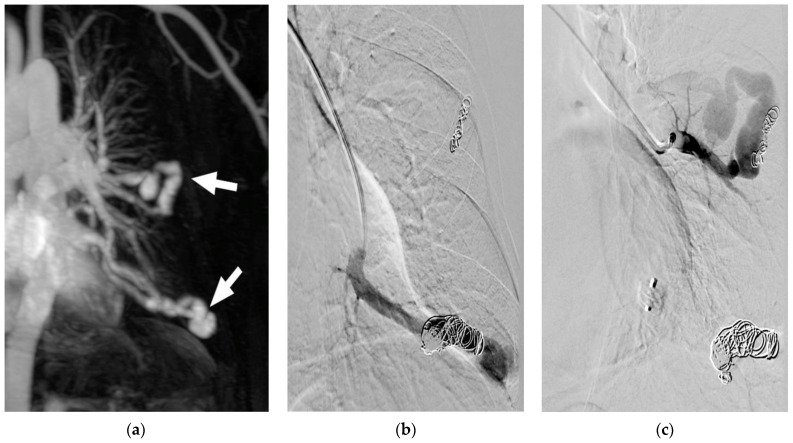
(**a**–**c**) Re-perfused PAVM after previous treatment elsewhere. Contrast-enhanced MRA (**a**) shows two large, re-perfused PAVMs (arrows) with early enhancement of the draining vein. In (**b**) the DSA of one re-perfused PAVM is shown, depicting insufficient dense packing of coils resulting in reperfusion of the vessel. No guide wire should be used, since small thrombi from the coils might be mobilized and lead to systemic emboli. In (**c**) the second re-perfused PAVM is demonstrated, showing only small coils at the wall of the vessel. Embolization was performed proximal to the treated vessel segment to avoid possible migration of the coils.

**Figure 4:**
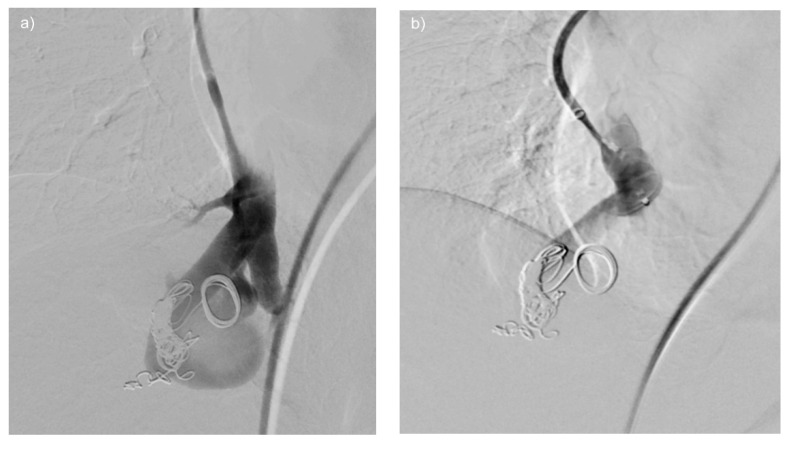

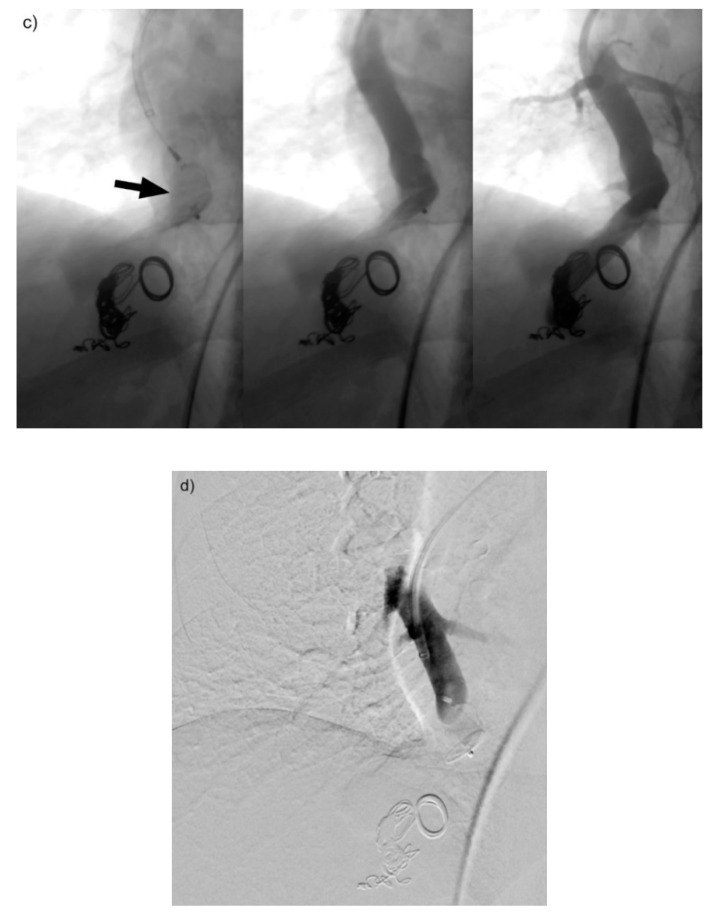
(**a**–**d**) A giant re-perfused PAVM in the lower right lobe. In this case, pre-interventional DSA (**a**) shows two large feeding vessels originating from a common trunk, resulting in embolization being performed at the level of the bifurcation. With DSA performed just after implantation, (**b**) shows the amplatzer plug II still connected to the wire. Optimal positioning is depicted. The dynamic series in (**c**) shows the vascular plug (arrow) still penetrable to contrast medium, however, flow is already reduced. At 5 min post-implantation of the vascular plug (**d**), the feeding artery of the re-perfused PAVM is completely occluded.

Clinical examples of diagnosis and treatment are depicted in Figure 1, Figure 2, Figure 3 and Figure 4.

## 4. Discussion

In general, all vascular interventions involving the thoracic or the supra-aortal regions bear the risk of clinically (in)conspicuous cerebral ischemic lesions, as reported for transcatheter aortic valve implantation or in carotid angioplasty [29,30]. Thus, all patients undergoing thoracic or supra-aortal interventions are prone to dementia and cognitive dysfunction [31,32]. Our prospective study on the occurrence of procedure-associated brain lesions following catheter-based embolization of PAVMs in patients with HHT suggests that this procedure carries a very low risk of cerebrovascular incidents in this patient population. Although our study can only be considered preliminarily, only one individual exhibited newly formed, clinically inconspicuous, small and diffuse cerebral emboli at 4 h after the interventional procedure, and this patient was unique amongst the patients in our cohort due to the presence of corroded tungsten coils from previous interventions. Embolization of the vessel had to be performed proximally and distally to the corroded coils and thus, when forwarding the catheter through the corroded coils, the risk of additional small displaced fragments was unavoidable.

To our knowledge, this is the first study to report on the incidence of peri-procedural cerebrovascular incidents following catheter-based embolization of PAVMs. Although the incidence of ischemic stroke ranges between 9 and 18% in patients with HHT and patent PAVMs [19,20,33,34,35], our findings suggest that embolization therapy does not significantly impact the rate of further cerebrovascular incidents. Indeed, the one event noted in our series can be ascribed to the embolization material used previously (tungsten coils) rather than to the embolization procedure itself. Given the relatively high number of participants and the high number of complex PAVMs warranting re-embolization therapy due to reperfusion, our results confirm that embolization therapy is safe and highly effective for the treatment of PAVMs in patients with HHT [36,37,38].

Available data regarding the occurrence of peri-interventional cerebrovascular incidents measured with DWI sequences in cMRI suggest that new ischemic brain lesions occur in up to 34% of patients treated for carotid stenting [23]. However, neurovascular interventions are more prone to acute cerebral embolism. Moreover, studies investigating the implementation of protection devices during carotid stenting have not shown statistically significant reductions in the incidence of acute peri-interventional embolism [30,39,40].

There were some limitations to this study. First, as in all interventional studies, evaluation of peri-procedural complications requires expertise and experience. This was a single-center study with procedures performed and assessed by only a few, very experienced interventional radiologists. It is possible that a greater number of cerebrovascular incidents might have occurred in this same patient cohort, had the interventions been performed by less experienced physicians. However, it should be noted that PAVMs are a complex pathology that warrant a certain degree of interventional experience by the treating physician, and that intervention would usually be undertaken at dedicated institutions by experienced personnel. Second, we only evaluated safety in terms of cerebrovascular incidents. Further studies are needed to evaluate the incidence of other potential complications, such as chest pain, hemoptysis, and hemothorax. Moreover, cMRI readings could not be blinded, since examiners were instructed to carefully look for new, small ischemic lesions in the brain, therefore being aware of the embolization therapy and patient’s disease.

In conclusion, to our knowledge, this is the first study to investigate the safety of catheter embolization of PAVMs in patients with HHT in terms of cerebrovascular incidents. Although further multi-center studies in larger patient populations are required to confirm our preliminary results, our observational study reveals a very low rate of clinically inconspicuous cerebral ischemia in patients with HHT undergoing interventional treatment for PAVMs.

## Data Availability

The data presented in this study are available on request from the corresponding author. The data are not publicly available due to privacy and ethical reasons.

## Abbreviations

HHT	Hereditary Hemorrhagic Telangiectasia
(P)AVM	(Pulmonary) Arteriovenous Malformation
AVP	Amplatzer Vascular Plug
cMRI	cerebral Magnetic Resonance Imaging
CE-MRA	Contrast Enhanced Magnetic Resonance Angiography
DWI	Diffusion Weighted Imaging
ADC	Apparent Diffusion Coefficient
MR(I)	Magnetic Resonance (Imaging)
GFR	Glomerular Filtration Rate
INR	International Normalized Ratio
DSA	Digital Subtraction Angiography
BW	Body Weight
TSE	Turbo Spin Echo
